# Cancer metastasis *in vitro* models – can 3D biofabrication and microfluidics sow the seeds in the right soil

**DOI:** 10.3389/fbioe.2026.1794850

**Published:** 2026-03-26

**Authors:** Yordan Sbirkov, Ilian Koev, Azad Ozanian, Meri Hristamyan, Judith Hagenbuchner, Michael Ausserlechner, Victoria Sarafian

**Affiliations:** 1 Department of Medical Biology, Medical University of Plovdiv, Plovdiv, Bulgaria; 2 Research Institute, Medical University of Plovdiv, Plovdiv, Bulgaria; 3 Department of Neurosurgery, University Hospital Pulmed, Plovdiv, Bulgaria; 4 Department of Epidemiology and Disaster Medicine, Medical University of Plovdiv, Plovdiv, Bulgaria; 5 Department of Pediatrics I and 3D Bioprinting Lab, Medical University of Innsbruck, Innsbruck, Austria

**Keywords:** biofabrication, cancer modelling, metastasis, microfluidics, organ-on-a-chip

## Abstract

More than 140 years after the first observations that cancer cells spread to secondary sites nonrandomly, the lack of representative pre-clinical models of metastasis precludes our understanding of the processes of metastasis. The development of new 3D biotechnologies, biomaterials, tissue engineering and more intricate *in vitro* experimental systems, however, can allow for the in-depth study of the main steps of metastasis–invasion, intravasation, circulation in the bloodstream, extravasation and colonization of new sites. In this review, we discuss the improvement of pre-clinical models with a focus on 3D biofabrication and organ-on-a-chip techniques. A systematic and critical description of the current models based on the most common sites of metastasis–the liver, the lungs, the brain and the bones is presented. The current progress in the development of the toolbox to study the mechanisms behind tumour spreading is provided. Several limitations and challenges are also highlighted with the goal to ultimately understand and prevent the major cause of cancer related deaths–metastasis. The convergence of microfluidic chip devices and bioprinting with micrometer precision, together with the implementation of biosensors measuring cellular parameters, can provide tools for the creation of vascularised multi-organ experimental systems. They reflect the complexity of human organs much more accurately than current models and can pave the way for personalised medicine and anti-metastatic drug discovery.

## 1 Introduction

Metastasis is the basic cause of death from any cancer after a period of dormancy lasting for months up to decades depending on the type of the tumour (Massague and Ganesh, 2021). It is a long and complex process involving numerous molecular mechanisms. The epithelial-mesenchymal transition (EMT) allows cancer cells to escape the basement membrane and to form secondary metastatic sites (Pastushenko et al., 2021). As soon as EMT cells invade metastatic tissue, metastatic cancer stem cells (MCSCs) at the tumour front infiltrate surrounding tissues via blood or lymph. MCSCs express thrombin, bind to platelet coagulation factors and activate SMAD and Notch pathways to preserve mesenchymal characteristics. Another player implicated in the metastatic cascade are the circulating tumour cells (CTCs) (Labelle et al., 2011). They attract platelets and neutrophils to escape anoikis and immune response. Once in secondary locations, cancer cells may become dormant to adapt to the new niche environment through EMT plasticity (He et al., 2023).

These first steps in tumour metastasis are common for the majority of cancer types including uveal melanoma (UM), the most common eye malignancy. Although it is a very rare cancer accounting for about 3400 cases per year (or about 0.0017% of all people diagnosed with cancer) (Siegel et al., 2024) it was exactly the work on UM that prompted the Austrian ophthalmologist Prof. Dr. Ernst Fuchs in 1882 to propose that cancer cells do not metastasise randomly, but have their “preference” for specific organs (since UM spreads most commonly to the liver) (E. et al.). The question of why cancer cells find the best conditions for development in the liver remains unanswered, especially considering the existence of circulating tumour cells which can practically reach any organ. The mechanisms behind this marked organotropism (e.g., UM, colorectal cancer (CRC) for the liver, breast cancer cells for the lungs and bones, and others) are not fully understood. Nearly 140 years ago, the British surgeon Stephen Paget, inspired by Fuchs, introduced the “seed and soil” hypothesis based on his observations from more than 700 autopsies of women with breast cancer (Paget, 1989). The theory that specific cells (“seeds”) need to reach a particular tissue/microenvironment (“soil”) that would support their growth away from the primary site of the tumour was confirmed in an experimental animal model of melanoma, which preferentially metastasises to the lungs. Kinsey confirmed this organotropism of melanomas *in situ* with experiments in mice where pieces of lung tissue taken from a newborn animal were implanted in their thighs. Metastases were found in both the lungs of the mice and the implanted lung tissue in the thighs (Langley and Fidler, 2011; Kinsey, 1960). Importantly, almost 140 years after the initial observations were documented, the molecular mechanisms, signaling pathways and especially the early steps of metastasis (the attraction of the “seed” to the “soil” and the “germination” of the “seed”) are not well understood.

Metastatic organotropism has been proved to depend on numerous factors like the layout of the circulatory system, the morphological and physiological characteristics of organs, intrinsic properties of the cancer, the organ-specific microenvironment, and the interplay between cancer cells and the immune system (Fan et al., 2021). Additionally, metabolic reprogramming, tumour-related genes, tumour-derived exosomes, and immune cell interactions also contribute to the organ-specific metastasis (Schild et al., 2018). The main five steps in the process of metastasis have long been described: invasion and migration; intravasation; circulation in the bloodstream; extravasation and colonisation in the new tissue (Brooks et al., 2024). Although well known, not all these steps can be studied in animal models or in standard 2D or 3D cell cultures (except for quasi-migration/invasion assays), let alone altogether as sequential events that follow each other. That is why it is necessary to develop more intricate experimental platforms like microphysiological systems (MPS), organ-on-a-chip (OoC) and bioreactors, that can allow *in vitro* metastasis research and can generate more accurate and predictive results from drug testing (Neufeld et al., 2022).

**FIGURE 1:**
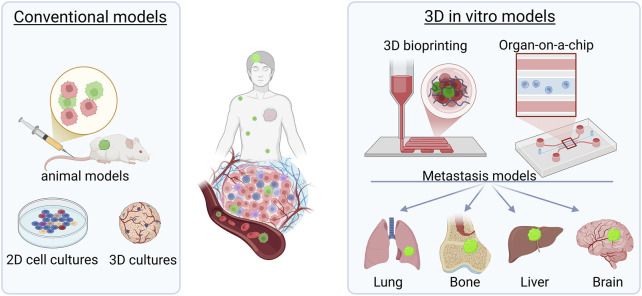
Summary of strategies for studying metastasis. Conventional models to study metastasis include animal models and 2D co-cultures of tumour and target tissue cells or 3D cultures of cell embedded in hydrogels. Novel 3D biofabrications methods take advantage of 3D bioprinting technologies and a variety of types of microfluidic organ-on-a-chip devices.

The present review summarizes the basic achievements in the field of *in vitro* modeling of cancer metastases with focus on the most common foci of secondary tumours (Figure 1). It highlights the importance of 3D biofabrication for disease modeling and personalized treatment.

## 2 Metastasis modeling strategies

**FIGURE 2:**
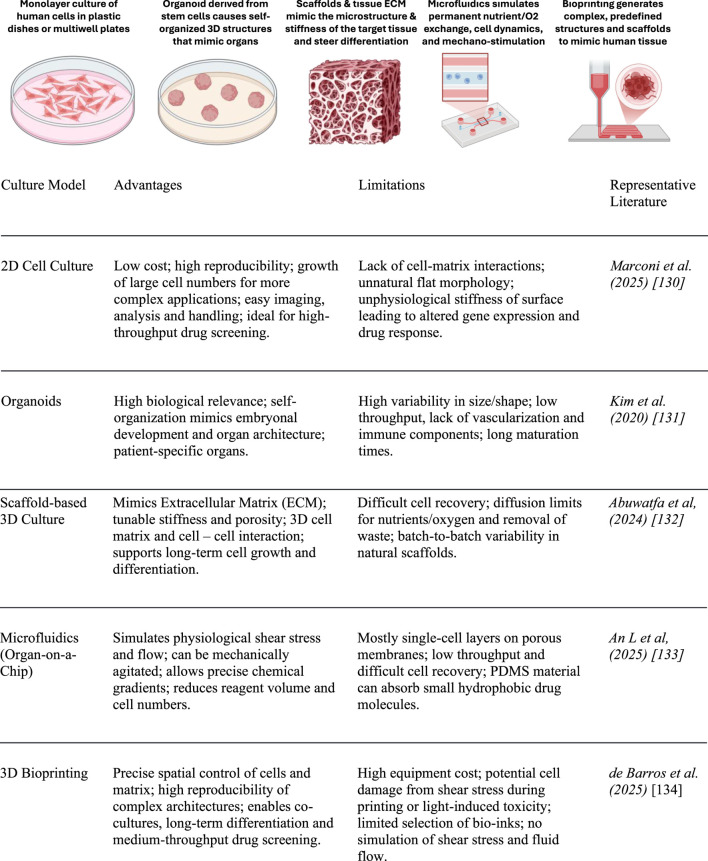
Comparison of *in vitro* culture methods in cancer research.

Various methods of culturing human cells have been developed (Figure 2). Many of these methods only replicate the situation in a human patient superficially in an overly simplified manner (2D cell culture) or partially (organoids, microfluidics and bioprinted scaffolds). This means that they represent a biased environment for studying metastasis. However, combining these methods—for example, using tissue-specific scaffolds structured via bioprinting in fluidic chip devices that support organ- and tissue-like cell differentiation—could provide a more realistic simulation of metastatic processes in complex organisms.

### 2.1 Standard 2D cultures

Although a number of techniques have been established for the study of proliferation, migration, cell adhesion, colony formation and for the elucidation of some molecular markers related to the EMT (Xiang et al., 2022), in practice, 2D cultures consider single aspects of a multi-component process. Thus, 2D models fail to address a number of factors that are involved in the complex pathogenesis of tumour metastasis like 1) tumour microenvironment, 2) remodeling of the extracellular matrix (ECM), 3) direct interaction between different types of cells, 4) paracrine action of signaling molecules, 5) role of the endothelium, 6) role of the immune system, 7) hypoxia, 8) cell metabolism, 9) neoangiogenesis and many others.

Although the liver, with its rich dual blood supply via the portal vein and hepatic artery, as well as its permissive microenvironment facilitating tumour cell colonization, is among the most common sites of metastatic spread, almost no 2D experimental models of liver metastases can be found in the literature. A PubMed search with the keywords “colorectal cancer (CRC)”, “liver”, “metastasis” and “Caco-2” or “HCT 116” (the two most used CRC cell lines) found only 15 and 13 results in the last 5 years, respectively. Most of the articles validated their findings in mouse models. One of the few examples comes from a simplified system using conditioned medium from liver endothelial cells that stimulates a CRC cell line to proliferate more rapidly and become more resistant to chemotherapeutics by activating the AKT signaling pathway (Wang et al., 2019). In another study with 2D cultures, Sakamoto et al. found that the Src kinase was responsible for HCT15 cell line adhesion. A role in cell metastasis was established at a later stage by implantation in the peritoneum of mice, illustrating the lack of appropriate *in vitro* 2D models (Sakamoto et al., 2001).

### 2.2 3D scaffold and scaffold-free systems

Multicellular tumour spheroids resemble the natural characteristics of metastatic solid tumours by producing cell aggregates with central necrosis due to lack of nutrients and oxygen. The outer zone contains proliferating cells (Grimes et al., 2014a; Grimes et al., 2014b). There are studies showing that 3D spheroids combined with 2D endothelial cells can form tubule-like structures that mimic vessel sprouting and angiogenesis (Chaddad et al., 2017). However, few examples of such vascularised models in the context of metastasis exist even if more complex 3D bioprinting and microfluidic approaches are integrated together (Nothdurfter et al., 2022). Combining multicellular tumour spheroids with relevant bioreactors allows the reconstruction of metastatic microenvironments representing the crosstalk within and between cells (Qiao and Tang, 2018).

Organoids are 3D-structured group of cells derived from primary tissue, embryonic stem cells, or pluripotent stem cells. They can undergo self-renewal and self-organization which reproduces tumour heterogeneity and microenvironment. Organoids mimic some, but not all, of the structures and functions of real organs and cannot recapitulate all the stages of cancer (Lancaster and Knoblich, 2014). Since not all cell types can form organoids and the fact that the latter lack vascularisation limits their suitability for modelling bone cancer metastasis. Furthermore, the possibility of growing matched normal and damaged organoids from patients permits screening of combinations of different drugs that specifically target the diseased tissue, reducing side effects.

3D scaffolds are 3D structures composed of different materials which mimic the microenvironment of specific tissues. Biological scaffolds consist of naturally derived ECM and are more similar to the physiologic microenvironment in terms of growth factors, cytokines, hormones, and other secreted molecules (Fang and Eglen, 2017). However, synthetic scaffolds provide higher reproducibility and superior control over biochemical and mechanical properties in 3D cell cultures compared to biological scaffolds. Due to their high-water content, the transport of oxygen, nutrients, waste, and soluble factors is facilitated (Mirkhala et al., 2023).

Recently, several 3D matrix-assisted assembly models of bone metastasis have been developed on both naturally and synthetically derived matrices, basically for prostate and breast cancer cell metastasis studies. The scaffolds used in these models enable metastatic cancer cell growth and mimic tumour microenvironment (TME) complexity (O'Rourke et al., 2017; Mohseni Garakani et al., 2022).

Garakani et al. created a 3D, compartmental tumour-stromal microenvironment model of patient-derived bone metastasis and proved by gene expression analysis that the 3D hybrid model showed EMT through increased expression of mesenchymal markers involved in the metastatic process. Therefore, tumour migration could be modelled *in vivo* and may be a suitable drug screening platform for personalized metastatic cancer treatment (Mohseni Garakani et al., 2022).

### 2.3 Animal models

As far as preclinical models are concerned, the main findings on the subject come from animal studies. There are several strategies that include the injection of tumours into the liver of rodents, cancer models with spontaneous metastases, or genetically engineered mice that develop primary tumours, which can then metastasise (Oh et al., 2017). In addition to the main drawbacks of animal studies, there are a number of other disadvantages to the use of such models. If the initial step of the metastatic cascade (invasion, intravasation, circulation in the bloodstream, extravasation and colonization) is considered, tumour invasion is highly dependent not only on the genetics and epigenetics of the malignant cells, but also on the microenvironment. In the case of xenograft models, the immune system of the experimental animal is markedly dysfunctional. This would not only compromise any conclusions regarding the ability of the tumour to invade the surrounding tissues, but will also cast doubts on weather and to what extend tumour cells would normally survive during the circulation and colonization steps in immunocompetent organisms. Therefore, poor tropism compared to patients is also an established disadvantage of xenograft models (Gomez-Cuadrado et al., 2017). If the second mouse modeling strategy is considered–injection or transplantation directly into the target tissue/organ, then none of the first steps of the metastasis cascade could be studied. If genetically engineered mouse models (GEMMs) are used, again there are several limitations. First, the time required for the development of the primary tumour may be very long and then for metastasis ь as well. Furthermore, the experiment may have to be terminated prematurely due to the primary tumour’s size (very few types could be removed to prevent this). Of note, GEMMs often rely on the (over)expression of oncogenes under tissue-specific promotors that may be difficult to regulate spatially and temporally despite ON/OFF molecular switch systems. Besides the challenging control of expression, this also means that GEMMs represent very specific hypotheses for the development of a particular type of cancer and do not recapitulate the variety of possible driving mutations in the clinic. Therefore GEMMs provide very limited representability and predictivity of the metastasis cascade and of drug response also. Moreover, even if spontaneous primary tumours develop and do not grow too much before metastases are observed, tissue tropism of tumour cell spread is limited and does not recapitulate the full picture observed in patients. For instance, breast cancer GEMMs would develop metastases in the lungs, but very rarely in the bones (Gomez-Cuadrado et al., 2017). Lastly, all of these experimental animal strategies cannot allow to monitor the steps of intravasation, tumour cell circulation, and extravasation in real time either.

In summary, important limiting conditions include 1) the time it takes for metastases to form, 2) the inability to trace the initial stages of the process, 3) the unnatural pathway of cell migration to the liver after injection of tumour cells, 4) premature termination of experiments due to excessively large primary tumours, 5) the cost of the experiments, 6) the limited number of laboratories suitable for this type of research, 7) the inaccessibility of models, and others (Oh et al., 2017).

Besides all abovementioned limitations, animal models and 2D cell cultures, have a more significant one–low predictability and translatability of the results to clinical practice.

### 2.4 *Ex vivo* models

*Ex vivo* models are comprised of freshly isolated tissue biopsies from patients. However, there is a smaller amount of data compared to primary tumours owing to the inoperability of a large percentage of metastases. Due to the preserved heterogeneity of the original tissue and the components of the surrounding microenvironment, these models are excellent platforms for personalizing therapy. *Ex vivo* explants are used to study the viability for long term cultures, analysis of tissue architecture, and response to therapies.

The *ex vivo* culture allows maintaining the interactions between tumour cells and the microenvironment. Other advantages include retaining the *in vivo* 3D distribution and the innate proportion of cells along with recapitulation of human bone conditions and finally, the less costly screening models. However, there are still several limitations that need to be considered such as: difficulty in obtaining tissue material and the need for optimization of the isolation and culture methods for each tissue type. Moreover, the models neither allow long-term monitoring of disease progression nor the examination of the long-term effect of drug treatments. Due to inter- and intra-patient tumour heterogeneity complex result interpretation is recommended (Laranga et al., 2020).

For example, Curtin et al. co-cultured over 7–14 days fresh human bone samples with human prostate cancer cell lines and showed that cancer cells formed independent tumour colonies similar to those present in human metastatic disease (Curtin et al., 2012). Tulotta et al. constructed an original model by mixing 3D *ex vivo* patient-derived xenograft, 2D *in vitro* breast cancer cells, and *ex vivo* bone specimens. The authors proved that after 4 weeks, bone implants were alive, re-vascularized, and remodelled. The expression profile of cancer cells identified molecular signatures which could serve as metastatic drivers predicting future relapse in bone in breast cancer patients (Tulotta et al., 2019).

### 2.5 3D bioprinting and microfluidic systems

Unlike 2D cell cultures, organoids and scaffold-based 3D cultures, 3D bioprinting enables the precise fabrication of complex tissue structures with defined spatial arrangements of channels and different cell types. Thus, a more natural cell morphology can be achieved, subsequently improving function and reproducibility. Furthermore, 3D (bio)printing methods can also facilitate the creation of larger structures, which rely on self-assembly and are more complex than organoids,. Microfluidics, on the other hand, overcome the problem of limited nutrient and oxygen diffusion in static cultures by providing continuous, controlled perfusion that mimics blood flow, which is essential for tissue maturation. Furthermore, multi-tissue interaction can be simulated by combining different tissue equivalents on microfluidic chips, enabling multi-organ systems - also known as ‘body-on-a-chip’ - and allowing researchers to study organ-to-organ communication, which is not possible in isolated organoids or conventional cell cultures.

#### 2.5.1 3D bioprinting

At its essence, 3D bioprinting in oncology can be defined as the layer-by-layer biofabrication of tumour constructs consisting of at least 2 components–living cells and supporting material (together referred to as a bioink), to recreate as many aspects of *in situ* cancer biology as possible. The capabilities of this technique offer a solution to several basic problems found in standard culturing methods: 1) the size and shape of the resulting tumoroids can be controlled, 2) their spatial distribution (for example, to obtain an interface between stromal or endothelial cells and cancer cells), 3) what substrate (component(s) of the ECM) the cells will contact, 4) the process of reproducibility, 5) the time it takes for tumour masses to form (it can take weeks for organoids, or months in animal models) (Kacarevic et al., 2018). There is a growing number of scientific publications with 3D bioprinted models of the most common types of cancer–breast, prostate, lung, ovarian, colorectal, glioma, etc. According to a review by Sztancovicz et al. from February 2023, there are a total of about 180 original publications on the subject (Sztankovics et al., 2023). Our PubMed search for “3D bioprinting” AND cancer identified more than 330 articles as per February 2026.

In the most up-to-date overview on the topic of 3D *in vitro* modelling of mCRC a 3D bioprinted hepatocellular construct that serves for research on mCRC was reported (Zhou et al., 2024). Sun et al. were able to isolate liver metastasis cells from 24 patients, print them in gelatin and algenate bioinks in 48-well plates, and test several chemotherapeutic agents alone and in combinations. The authors found differences in sensitivity to 5-fluorouracil and oxaliplatin between primary tumours and metastases, as well as an excellent correlation between drug response *in vitro* and clinical progression of the disease (Sun et al., 2024). The results were so promising that one of the clinics in Beijing has started a clinical trial (NCT04755907) to personalise the therapy for mCRC using the developed experimental algorithm. This research proves the undeniable advantages of 3D bioprinting over all other preclinical methods, but the focus is on the metastases themselves, and not on the interactions between tumour cells and hepatocytes, or on the processes that lead to the development of metastases.

#### 2.5.2 Microfluidics and organ-on-a-chip

**FIGURE 3:**
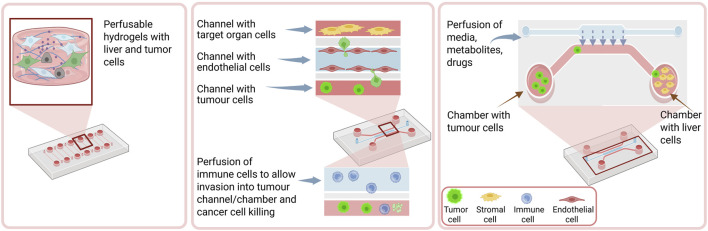
Concepts of microfluidic organ-on-a-chip systems. Left - Simpler microfluidic organ-on-chip-systems can consist of perfusable chambers that contain cells in 2D or embedded in hydrogels (left). Middle - Most chips would include compartments that are connected with microchannels, where cells can be infused (injected) and are also separated by membranes that would allow cell migration from the channels to the chambers. Right–Several chips contain multiorgan chambers where different types of cells are seeded and connected to each other through microchannels so tumour cells can migrate from one compartment to another “organ” chamber.

Microfabrication techniques and microfluidic technologies help the creation of microstructures able to manipulate small amounts (10^−9^ to 10^−18^ L) of fluids. The microfabricated channels can accommodate different kinds of cells and tissues with controllable shape and function. They can also allow dynamic fluid flows and spatiotemporal gradients to be controlled in a 3D culture microenvironment (Kim et al., 2023). Microfluidic-based fibrous tumour tissues include fibers, tubes, and filaments and recreate better biomimetic microenvironments *in vitro*. They can incorporate vasculature or spatially differing matrix properties to allow cancer cell migration and invasiveness (Figure 3)**.** Microfluidic devices can also be integrated with a 3D bioprinter to produce multiphasic bone metastasis tissue models (Khanmohammadi et al., 2025).

By *in vitro* combining organoids and microfluidics organ-on-a-chip models are constructed which are valuable tools to examine tumour growth, angiogenesis, migration, metastasis, and drug response. These devices consist of both cells and ECM, allow a precise control of the microenvironment and continuous flow perfusion (Kim et al., 2023). They can also mimic the TME with its multicellular interactions, ECM-based biochemical properties, biophysical signals and their gradients (R et al., 2023).

### 2.6 Cell type considerations when developing *in vitro* models

The selection of cell types that are representative of the desired pathological condition is a critical step in the development of physiologically relevant and predictive metastasis experimental platforms. In the vast majority of published work discussed later, the research teams take advantage of established cell types with defined genetic, phenotypic and functional characteristics and optimised growth conditions. Cell lines, however, lack the heterogeneity observed in patient tumors. Therefore, many groups would use several cell lines and would observe different results (e.g., two or three breast cancer cell lines exhibiting distinct organotropism) (Firatligil-Yildirir et al., 2021; Liu et al., 2025). Primary cells would best represent tumor heterogeneity. Nevertheless, adapting patient-derived cells or organoids (PDOs) to any experimental system is very challenging since, even if malignant, they may not be grown for extensive periods of time which limits the number of assays that can be performed. Liu et al. have shown that primary breast cancer cells that are resected from lung metastasis indeed migrate preferentially to lung fibroblasts (as opposed to, e.g., hepatocytes), but also pointed out that there cannot be any statistical verification of the results if obtained from a single PDO in a single experiment (Liu et al., 2025).

For a representative metastasis model, multiple cell types have to be incorporated in the same experimental platform. Thereby, heterotypic co-cultures are required which poses a number of technical challenges (e.g., different growth rates and culture media), but also a set of questions that need to be addressed. The main issues are how to control and mimic their natural geography/architecture in the target organ. It is also important to consider what cell numbers and ratios between the cell types would be physiologically relevant at time point zero and over the course of the experiments when different cell types would proliferate differently. Another concern is to recreate and maintain the functionality of the target organ/tissue and to incorporate successfully endothelial and immune cells (Childs et al., 2022). Therefore, a few compromises would have to be made in any experimental approach to make it fit-for-purpose with respect to the particular hypotheses and questions that the experiments aim to address.

## 3 Applications of 3D bioprinting and organ-on-a-chip technologies by site of metastasis

### 3.1 Brain

Metastatic disease is considered as one of the primary reasons for cancer-related death, with brain metastasis being the most malicious process as generally patients with brain metastases from extracranial primary tumours have a limited survival. It is often within the range of 3–9 months (Nieblas-Bedolla et al., 2022; Nieder et al., 2022). The overall 1-year survival rate is about 20%–40%, depending on the primary cancer type, the topology and the extent of metastasis. Three out of four brain metastases diagnosed in the clinic are derived from lung cancer, breast cancer and melanoma, in this order of incidence (Masmudi-Martin et al., 2021).

General population-based epidemiology studies reported brain metastasis incidence rates of 2.8–14.3 per 100,000 people. Cancer registry data indicate that 5.3%–9.6% of all newly diagnosed cancer patients will develop brain metastasis (Yuzhalin and Yu, 2020). However, this data is probably undervalued because of the infrequency of autopsies in patients who die from metastatic disease and the lack of recommendations for routine brain magnetic resonance imaging in patients without neurological symptoms. Of note, metastases to the brain are much more frequent than primary brain malignancies such as gliomas (Davis et al., 2012), which may be due to the low number of dividing cells in the brain.

The heterogeneity of the patient population turns brain metastases into a complex challenge as they may arise from a wide variety of primary tumours. Furthermore, patients may have previously received several treatments for their primary cancer, and/or have developed resistance to multiple lines of therapy. The availability of optional treatments in combination or alone make brain metastasis a serious hurdle (Sperduto et al., 2020).

Tumour cells exhibit a variety of specialized mechanisms to penetrate the blood-brain barrier (BBB) and preferentially seed into the brain. A major obstacle in all brain biofabrication approaches is the complexity of the BBB which is made up of 4 cell types - pericytes, neurons, astrocytes, and brain endothelial cells with tight intercellular connections and provides selective permeability of molecules and cells between the blood and brain tissue (Yamamizu et al., 2017; Abbott, 2013). Therefore, modelling the brain architecture and the BBB is crucial for the *ex vivo* mimicking of brain metastasis. The process of metastasis in the brain occurs in two consecutive and interrelated stages: transendothelial extravasation through the BBB, and subsequent colonization in the brain parenchyma (Evangelou et al., 2025).

The high cellular and molecular complexity of the BBB requires the development of intricate *in vitro* systems such as microfluidic brain-on-a-chip and hiPSC-based microfluidic systems. These models aim to mimic the cellular architecture, neuroglial communications, the selective barrier function of the BBB, and the dynamics of brain metabolism in order to reliably simulate all key stages of the brain metastasis process (Yamamizu et al., 2017; Abbott, 2013).

Microfluidic brain-on-a-chip is a powerful technology to study brain functions in normal and pathological conditions (Mofazzal Jahromi et al., 2019). A 3D microfluidic device containing astrocytes, neurons, and human cerebral microvascular endothelial cell (hCMEC/D3) showed that the endothelial cells monolayer acts as a size-selective barrier *in vitro* similar to a functional *in vivo* BBB (Ad et al., 2017). Another microfluidic BBB model containing brain endothelial cells derived from iPSCs demonstrated trans-epithelial electrical resistance (TEER) values of up to 4000 Ω*cm^2^ and mimicked the physiological function of the BBB for both large molecules, such as fluorescein isothiocyanate (FITC)-dextran, and small model drugs, such as caffeine. This highlights the importance of microfluidic BBB systems in studying BBB transitions (Wang et al., 2017).

The hiPSC-based microfluidic systems can be useful tools to study not only neurodevelopmental diseases but metastasis to the central nervous system as well. Neural cells derived from hiPSCs may serve as a dynamic model to evaluate the functional activities of the human brain also in the process of metastasis (Qian et al., 2018). However, there are several technical challenges to be tackled by microfluidic brain-on-a-chip systems. A major concern is the tendency of drugs and chemicals to bind non-specifically to PDMS. Additionally, avoiding bubbles, different flow rates between platforms, optimal hemoglobin-based oxygenation and nutrient levels, and inclusion of biosensors create problems during the manufacturing process (Low and Tagle, 2017). Further difficulties include the modelling of cell–matrix or cell–cell interactions, cell migration, and 3D cell growth especially of different cell types while mimicking brain metastasis (Mofazzal Jahromi et al., 2019).

Aleman and Skardal developed a metastasis-on-a-chip device, that houses multiple bioengineered 3D organoids, established by a 3D photopatterning technique and ECM-derived hydrogel biomaterials. A CRC organoid was placed in a single microfluidic chamber connected to multiple downstream chambers in which different organ constructs were housed. Under recirculating fluid flow, tumour cells entered the circulation and preferentially homed to these tissue constructs to which the primary cancer cells are most frequently seeded in human patients (Aleman and Skardal, 2019).

Xu et al. fabricated a multi-organs-on-a-chip platform to investigate lung cancer metastasis to the brain, bone, and liver. They cultured bronchial epithelial, lung cancer, microvascular endothelial, mononuclear, and fibroblast cells divided by membrane in the “lung” chamber, while astrocytes, osteocytes, and hepatocytes were cultured in distant chambers, mimicking the metastatic process of lung cancer cells in all these organs. Lung cancer cells formed a “tumour mass” in all three “organs” showing signs of EMT and also induced astrocyte damage highlighting the relevance of this model to study brain metastasis (Xu et al., 2016).

Recently, the cancer-on-chip technology was combined with advanced live cell imaging algorithm and artificial intelligence. Oliver et al. developed a platform for artificial intelligence-based identification of the extravasation potential of cancer cells into the brain metastatic niche. They combined AI, a BBB-on-a-chip and confocal tomography to discriminate between the metastatic signatures of cancer cells (Oliver et al., 2019). This chip allows to detect the migratory and proliferative phenotypes of cancer cells with different degrees of brain metastatic potential. It also discovers cells from cancer patient samples with known metastatic potential. Using AI to complement the cancer-on a chip models, it is possible to predict the metastatic capacity of cancer cells which could be a valuable contribution to clinical practice in oncology.

### 3.2 Bone

Bones are one of the most common sites for distant metastases in advanced solid tumours due to their unique microenvironment, which is rich in blood vessels and has a dynamic remodeling cycle (Jayarangai et al., 2025). Breast cancer is the most common cause of bone metastases. In advanced disease, 65%–80% of patients develop skeletal metastases, usually osteolytic (15%–20%), osteoblastic or mixed. In prostate cancer, bones are the most common target for metastatic lesions, which are almost exclusively osteoblastic, modulated by endothelin-1, PSA, and Wnt/β-catenin/RUNX2 activation (Macedo et al., 2017; Deng et al., 2025). Lung carcinoma predominantly causes osteolytic metastases in 30%–40% of cases, mediated by matrix metalloproteinase-13 (MMP-13) and Parathyroid hormone-related protein (PTHrP)-dependent osteoclast differentiation (Jayarangai et al., 2025; Macedo et al., 2017). CRC bone metastases are not very common, with incidence varying from 1.2% to 12% and a ten-year incidence of about 2.7% (Holladay et al., 2023; Park et al., 2020; Li et al., 2022). More recent and larger investigations report bone metastases in 6%–10% of CRC patients, reflecting improved detection and longer survival (Holladay et al., 2023; Matias-Garcia et al., 2024; Ilyas, 2024). Autopsy studies suggest higher rates (up to 24%–27%), likely due to the detection of asymptomatic cases (Matias-Garcia et al., 2024; Ilyas, 2024). Bone metastases are more frequent in patients with rectal cancer and those with underlying lung or liver metastases (Holladay et al., 2023; Matias-Garcia et al., 2024). They typically occur late and almost always in the presence of other visceral metastases (Holladay et al., 2023; Matias-Garcia et al., 2024; Ilyas, 2024) and are associated with skeletal-related events (Park et al., 2020).

Metastases in the bones are also found in other solid tumours such as kidney cancer, thyroid cancer, bladder cancer, multiple myeloma, melanoma, and sarcoma. Each neoplasm causes different lesions with a characteristic osteolytic or osteoblastic profile. Prognosis is poor, with median survival of 7–9.4 months after diagnosis for populations where treatment may be less aggressive or patients present with extensive disease and more comorbidities (Park et al., 2020). In cohorts where patients undergo more aggressive treatments such as metastasectomy and systemic therapies median survival can be approximately 17.8–18 months, reflecting a subset of patients with better prognosis. The 5-year survival remains low, roughly around 5.7%, indicating that long-term survival after bone metastasis is rare (Holladay et al., 2023).

When recreating the the bone microenvironment so that metastasis to this site can be modelled *in vitro*, certain biological characteristics of this niche need to be considered. Bone tissue is a dynamic system in which osteoblasts, osteoclasts, and osteocytes maintain a constant remodelling balance. The latter is regulated by a complex network of cytokines, growth factors, and hormones. Bone marrow is richly vascularized, which facilitates the retention and extravasation of circulating tumour cells. The release of growth factors such as transforming growth factor-beta (TGF-β), insulin-like growth factor (IGF), and platelet-derived growth factor (PDGF) during bone resorption stimulates tumour cell proliferation. The “seed and soil” hypothesis explains the affinity of some tumours for bone by a match between the biological needs of tumour cells (“seeds”) and the favourable bone microenvironment (“soil”) (Elshimy et al., 2025).

Bone metastases can be osteolytic (common in breast and lung cancer) or osteoblastic (common in prostate cancer), with many metastases being mixed in nature. The RANK/RANKL/OPG axis plays a key role in the process. RANKL (secreted by osteoblasts and stromal cells) activates osteoclasts, leading to bone resorption and the release of growth factors. TGF-β signaling stimulates the expression of PTHrP in tumour cells, enhancing osteoclastogenesis. The chemokine axis CXCL12/CXCR4 directs tumour cells to the bone marrow. Exosomes and microvesicles carrying miRNA and proteins remodel the bone niche. Metabolic adaptations include the utilization of lipids and glutamine, as well as adaptation to the hypoxic environment of the bone marrow (Elshimy et al., 2025).

The complex bone microenvironment imposes the search for new strategies to mimic the 3D cellular architecture. There are several 3D scaffold fabrication techniques which reconstruct the *in vivo* process of bone metastasis (O'Rourke et al., 2017; Mohseni Garakani et al., 2022). Along with these approaches, microfluidics, organ-on-a-chip, and 3D bioprinting models are also promising strategies to rebuild the bone microenvironment and the interactions between it and metastasizing cancer cells.

Hao et al. designed a bone-on-a-chip microfluidic device that mimics bone metastasis. The authors combined breast cancer cells and osteoblastic tissue. Genetically altered metastasis-active breast cancer cells disrupted the osteoblastic tissue, whereas the metastasis-suppressed cells remained dormant (Hao et al., 2018). Mastro et al. used a bioreactor-based model with mineralized osteoblast tissue to show that dormant cancer cells could be reactivated by bone remodelling cytokines such as TNFα and IL-1β, mediated by prostaglandins. When the prostaglandin pathway was suppressed, this transition was blocked (Mastro and Vogler, 2009).

Three-dimensional bioprinting technologies are also useful in studying angiogenesis, invasion and metastasis. To investigate breast cancer bone metastasis, Zhu et al. combined 3D bioprinting and biomaterials and produced *in vitro* bone matrices composed of PEG hydrogel and different concentration of nanocrystalline hydroxyapatite. They used a stereolithography-based 3D printer to fabricate a bone matrix with finely tuned architecture which was utilized to analyze the interaction between cancer cells and osteoblasts. The presence of breast cancer MDA-MB-231 cells affected the morphology, proliferation rate, and cytokine secretion of osteoblasts increasing their IL-8 secretion (Zhu et al., 2016). Several recent studies also proved the reliability of 3D printed bone matrices to evaluate bone metastasis (Sanchez-de-Diego et al., 2025; Lamouline et al., 2023; Conceicao et al., 2022).

### 3.3 Liver

The liver is the most common site of metastasis and a large meta analysis showed that more than 5% of all cancer patients present with synchronous liver metastases at diagnosis. The 1-year survival in such cases drops to only about 15%. The most frequent tumours that spread to the liver are breast cancer and CRC, but stomach, oesophageal, pancreatic cancers as well as melanomas are also frequently found to metastasise to the liver in older patient (Horn et al., 2020).

For CRC alone, 15%–25% of patients present with synchronous liver metastases at diagnosis. Overall, up to 50% develop liver metastases during their disease course even after initial treatment (Griscom and Wolf, 2025; Lin et al., 2025; Kow, 2019; Filoni et al., 2023; Ros et al., 2023) - in 25% they are detected at diagnosis, and in the remaining patients (18%–25%) metastases occur up to 5 years after the initial detection of the disease (Kow, 2019; Cheng et al., 2024). The age-standardized incidence rates for synchronous liver metastases are 7.6 per 100,000 (males), and 3.7 per 100,000 (females) (Manfredi et al., 2006). The pathophysiological mechanism can be explained with the fact that the portal vein system directly connects the colorectal region to the liver (Kow, 2019; Zhou et al., 2022). Risk increases with advanced tumour stage: 3.7% (stage I), 13.3% (stage II), 30.4% (stage III) (Manfredi et al., 2006). Liver metastases are the leading cause of CRC-related mortality (Lin et al., 2025). Furthermore, they have a significant negative impact on overall survival, with less than 30% survival rate within the 1st year (Manfredi et al., 2006). Median 5-year survival is less than 14% with palliative chemotherapy, but can reach 42% at 5 years after surgical resection (Filoni et al., 2023; Ros et al., 2023). Treatment includes surgical resection, ablation, and systemic therapy. Surgery offers the best chance for long-term survival (Filoni et al., 2023; Ros et al., 2023), but most patients are not eligible for curative surgery, hence palliative chemotherapy is standard for unresectable cases (Griscom and Wolf, 2025; Manfredi et al., 2006). In cases that are not operable even after aggressive courses of chemotherapy, 5-year survival rate drops to 10% (Kow, 2019).

The reason why tumour cells grow so frequently in the liver is at least partially due to blood flow and the mechanical entrapment of circulating tumour cells (CTCs) in this particular organ, but also to the specific microenvironment that hepatocytes create, which favors cancer cell survival and growth. A number of studies have investigated the pro-metastatic niche of the liver. An investigation on pancreatic cancer demonstrates elevated STAT3 signaling in hepatocytes that ultimately leads to IL-6 and serum amyloid A1 and A2 (SAA) secretion, subsequent recruitment of myeloid cells and fibrosis, all of which present a good “soil” for tumour cells (Lee et al., 2019).

**FIGURE 4:**
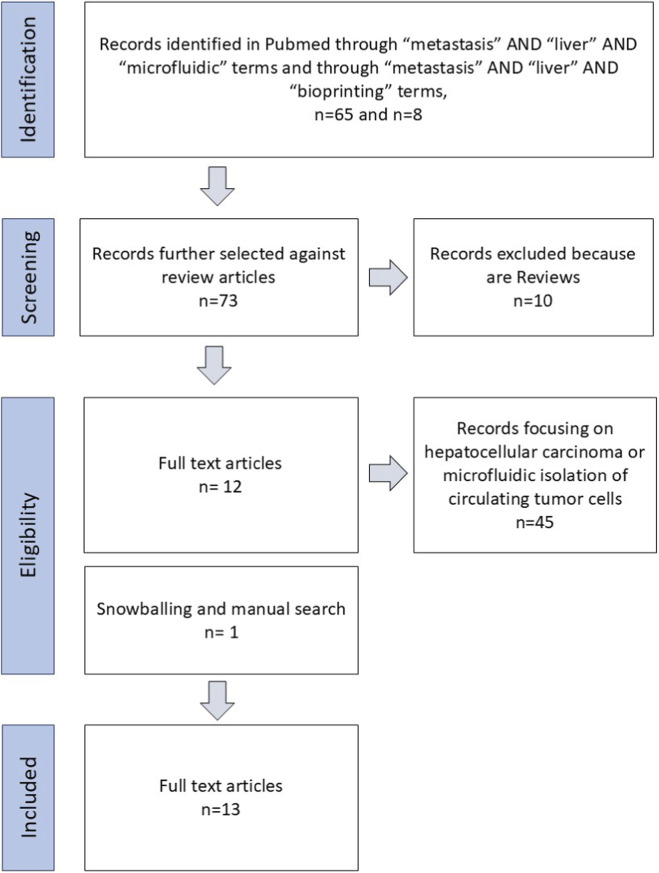
PRISMA flow diagram to identify research articles focusing on biofabricated liver metastasis models.

Nevertheless, experimental work related to liver metastasis is exclusively focused on animal models, which limits and slows down the progress of scientific discoveries in the field. Therefore, it is necessary to develop and validate experimental platforms/systems that allow 1) conducting *in vitro* studies to better understand metastasis processes and 2) predictive preclinical testing of new therapies for metastatic tumours. Of note, only a single microfluidic system of interaction of CRC tumour cells with endothelium is reported, but it lacks hepatocytes, therefore, it is not going to be discussed in this review (Strelez et al., 2021). All other relevant models that are identified after a systematic review (Figure 4) are described in the following section and summarised in Table 1.

**TABLE 1:** Summary of liver metastasis models.

Liver metastasis model	Cell used	Key features	Key findings	Reference
Prostate Breast	DU-145 Primary breast cancer Primary rat hepatocytes	Perfusion system of 1 chamber and seeded/flowing tumour cells monitored by 2-photon microscopy	Tumour cells would grow only within the 3D primary liver tissue	(Yates et al., 2007)
Breast	Primary human liver cells MDA-MB-231, MCF-7, and BT474	Development of LiverChip (Zyoxel Ltd., Oxford, UK); inlet micropumps for dosing insulin/glucagon, glucose/fructose, cortisol, and chemotherapeutics in the media	Breast cancer cells enter dormancy when cultured in the liver metastasis model	(Wheeler et al., 2013; Wheeler et al., 2014)
Breast	Primary human liver cells MDA-MB-231	LiverChip with PEG-fibronectin peptide perfusable hydrogel compartment to support 3D growth of breast cancer cells	Hydrogel niche recreates better the liver microenvironment; doxorubicin and cisplatin resistance	(Clark et al., 2016)
Kidney	Caki-1 HepLL	GelMA hydrogel with decellularized liver matrix containing both cell types at different ratios	Testing of PLGA-PEG nanoparticles for 5-fluouracil delivery shown more efficacious than 5-FU alone	(Wang et al., 2020)
Lung	A549 lung cancer HFL-1 lung fibroblasts L02 fetal liver cells	Two chambers (lung and liver) and microfluidics with hypoxia and normoxia gases	HIF-1a is elevated in hypoxic conditions and enhances metastasis through Snail 1 and 2	(Zhen et al., 2021)
Colorectal	HCT-116 Human intestinal epithelium (INT-407) Hep-G2 hepatoma	A “gut” compartment with tumour cells that would migrate to a “liver” compartment through the microfluidics of the chip	Validation of the system through perfusion of 5-fluouracil	(Skardal et al., 2016)
Breast	HUVEC endothelial cells MDA-MB-231, MCF7 breast cancer ACC-M salivary gland Primary rat lung, liver, bone cells	CXCL-12 stimulation of metastasis; metastasis chambers with primary rat cells and tumours cells flowing over them in endothelium-seeded channels	Attachment of tumour cells to endothelial and migration into metastasis chambers with lung, liver and bone cells	(Kong et al., 2016)
Lung	lung epithelial (A549), endothelial cells, fibroblasts, mononuclear cells and lung cancer cells astrocytes, osteocytes and hepatocytes	A multi-organ chip providing 3 sites of metastasis (brain, bone, liver) that were downstream of a lung cancer compartment with 5 cell types	Tumour formation in the three metastasis sites	(Xu et al., 2016)
Breast	breast epithelium MCF-10A, lung fibroblasts BRL-3A, rat liver cells BRL-3A HUVEC MCF-7 and MDA-MB-31	Two chips used – for invasion into breast, lung and liver cells in Matrigel, and for extravasation through a layer of endothelial cells	Different behavior of 2 breast cancer cell lines in terms of tissue tropism and extravasation	(Firatligil-Yildirir et al., 2021)
Colorectal	HepG2, A549, HUVEC HCT-116	5 chamber microfluidics chip where cells are embedded in a photocrosslinked gelatin-HA based hydrogel with ECM components	Preferential metastasis to liver and lung	(Aleman and Skardal, 2019)
Breast	LO2, h-MSCs, and HFL-1 MCF-7, MDA-MB-231, and SKBR-3 Patient samples	Chambers with fibrin gel mimicking lung, liver and bone tissues	Organotropism proved by metastatic patient sample selectivity for the lung chambers of the chip	(Liu et al., 2025)
Breast	MDA-MB-231; Human primary liver cells (HUM182641)	GelMA-based bioink with hepatocyte extracellular vesicles and tumour cells was 3D bioprinted and perfused	Lentiviral transformation of tumour cells into induced hepatocyte-like cells reduces metastasis in the chip platform	(Lu et al., 2023)
Breast	Liver endothelial cells	Breast cancer-derived extracellular vesicles used to study the premetastatic liver niche	EVs found to induce EMT and upregulate CXCR4 on endothelial cells, thus promoting metastasis	(Kim et al., 2020a)

The first experimental platforms for complex co-culturing of liver and malignant cells are more than 15 years old and are focused on prostate (Yates et al., 2007; Benam et al., 2015) and breast cancers (Yates et al., 2007; Wheeler et al., 2013; Wheeler et al., 2014). In 2007, Yates et al. were one of the first groups to recreate the liver as the site of metastasis through a perfusion organ-on-a-chip system consisting of primary rat hepatocytes expressing green fluorescent protein (GFP) and prostate cancer cells or primary mammary carcinoma cells labeled with red fluorescent protein (RFP). This experimental set up allowed for the dynamic following of the processes of adhesion and micro-tumour formation *in situ* by 2-photon microscopy. Interestingly, once tumour cells would establish growth within the primary rat hepatocytes, they would not be able to be cultured under standard conditions (even in a co-culture with hepatocytes) demonstrating the critical role of the liver microenvironment (Yates et al., 2007).

Several years later, another perfusable experimental platform was established (and commercialised - LiverChip (Zyoxel Ltd., Oxford, UK)) taking advantage of a chamber with primary human hepatocytes and the ability to introduce metabolites and chemotherapeutics in that closed system. Using this chip, Wheeler et al. found that a significant proportion of breast cancer cells enter the inactive phase (quiescence) when cultured in the 3D micromass of liver cells in the perfusable chamber (Wheeler et al., 2014). The same group developed another type of chip enhanced with a PEG-based hydrogel modified with fibronectin peptides for better cell adhesion. It was able to support 3D growth and liver micronenvironment ques for breast cancer cells. Not only did the researchers find that the hydrogel provides a more physiologically relevant environment for the functionality of hepatocytes (response to inflammatory stimuli, growth, metabolic function, drug response), but also the 3D co-culture would lead to breast cancer cell dormancy and resistance to cisplatin and doxorubicin (Clark et al., 2016).

More simplified strategies include co-culturing of tumour and liver cells as separate monolayers connected through microfluidics channels or all cells could be cultured together in 3D hydrogels. The latter has been implemented by Wang et al. who have used a kidney cancer cell line and hepatocytes embedded simultaneously (at different ratios) in GelMA-based hydrogels that were modified with decellularised ECM components from rat liver. The research group used their experimental set up to perfuse chemotherapeutics through these heterotypic hydrogels mimicking metastasis and validated PLGA-PEG nanoparticles as effective carriers of 5-FU (Wang et al., 2020).

In 2021, Zheng et al. developed a more complex organ-on-a-chip system. The research group used two chambers–one with fetal liver cells (L02 cell line) and another chamber with lung fibroblasts (HFL-1) and a lung cancer cell line (A549), to study how hypoxia affects metastasis. Implementing this novel chip system, HIF-1a was demonstrated to enhance EMT through elevation of Snail 1 and 2. Importantly, using HIF-1a inhibitors was shown to reduce *in vitro* metastasis in this model (Zhen et al., 2021). A similar concept with two chambers was implemented by Skardal et al. who in 2018 established a chip consisting of gut cells and CRC cells embedded in hydrogel that was connected to another organoid compartment with liver cells in a hydrogel. This system allowed for the monitoring of tumour cells that would migrate to the 3D liver structure and grow there in a metastasis-like manner (Skardal et al., 2016).

A multi chamber breast cancer metastasis chip microfluidics system has been developed so that tumour cells can flow in endothelium-seeded channels and migrate into the different chambers that would mimic lung, liver or bone (with muscle as control). Even though the sites of metastasis were recreated by using primary rat cells rather than human cells, Kong et al. managed to demonstrate that different cell lines have distinctive behavior in terms of number of metastasizing cells. Importantly, in this experimental set up, CXCL-12 was used to stimulate metastasis and an antagonist of its receptor (CXCR4) was capable of reducing the amount of metastatic cells (Kong et al., 2016). A similar concept, again in 2016, was introduced by Xu et al. who described another “multi-organ” chip consisting of an compartment with a co-culture of 5 types of cells–lung epithelial, endothelial cells, fibroblasts, mononuclear cells and lung cancer cells, that was connected by three channels to three other compartments that were the sites of metastasis–brain, bone and liver (containing astrocytes, osteocytes and hepatocytes, respectively) (Xu et al., 2016). The same type of approach with several different tissue types on the same chip has been adopted by Firatligil-Yildirir et al., in 2021, where lung, liver and breast chambers with the respective cell types embedded in Matrigel were used to follow the invasion of breast cancer cell lines. This invasion/chemotaxis chip allowed for the demonstration that MDA-MB-231, but not MCF-7 cells would invade the lung and liver micronenvironments. The same group used another type of chip (an extravasation chip) having a layer of endothelial cells and showed preference for migration of tumour cells towards the lung chambers (but extravasation towards the liver compartment was also observed) (Firatligil-Yildirir et al., 2021).

Again, investigating tissue tropism, Aleman and Skardal designed a 5-chamber microfluidics device where CRC cells from one chamber are infused towards the other 4 compartments containing endothelial, lung, liver and control (that was hydrogel only). Importantly, cells were embedded in a gelatin-HA-based hydrogel with ECM components to provide a 3D scaffold for the growth of the target tissues. In accordance with other studies, preferential homing of circulating HCT-116 cells to liver and lung was described (Aleman and Skardal, 2019). Similarly, in 2025 by Liu et al. with the aim to study organotropism of three breast cancer cell lines established liver, bone and lung chambers from LO2, h-MSCs, and HFL-1 cells. They were grown in fibrin hydrogels for 3 days prior to infusion of the tumour cells through microchannels that allowed connection between all chambers. Thus, tissue tropism was followed and it was found that the 3 different breast cancer cell lines exhibited different preferences (e.g., MCF-7 cells would not invade the lung fibroblasts as much as the other 2 cell types). Thus, the clinical relevance and predictivity potential of this *in vitro* model of metastasis and organotropism was validated (Liu et al., 2025).

Another approach to the development of liver-on-chip (LOC) systems has been adopted by Lu et al. who have established a perfusable 3D bioprinted chip with breast cancer cells. They mimicked the liver microenvironment with primary hepatocyte extracellular vesicles (EVs) and a GelMA-based hydrogel. The embedded MDA-MB-231 cells were successfully reprogrammed with the help of lentiviral transformation with six transcription factors into induced hepatocyte-like cells, which markedly reduced their cancer phenotype and metastatic growth in the LOC (Lu et al., 2023). The importance of EVs in the TME has been studied from a different angle as well. Using another LOC system, breast cancer-derived EVs have been shown to promote EMT and CXCR4 expression in liver endothelial cells through TGFβ1, thereby promoting metastasis (Kim et al., 2020a).

### 3.4 Lungs

The lung is the second most frequent site for metastasis from numerous types of cancer. About 20%–54% of malignant tumours may have pulmonary metastasis (Stella et al., 2019; StatPearls. https; Gerull et al., 2021). The most common ones that frequently metastasize to the lungs are: breast, colorectal, kidney, head and neck, and testicular cancers, as well as melanoma. However, cancers of the bladder, ovary, thyroid and pancreatic glands, and sarcoma can also spread to the lungs.

Lungs are the second most common site for CRC metastases, as they occur in about 10%–15% of patients (Ilyas, 2024; Beckers et al., 2021; Petrella et al., 2024; Guo et al., 2025; Han et al., 2022). Even though isolated lung metastases are quite uncommon (2.8%–7.4%) most patients with lung metastases have advanced disease and often exhibit other metastatic sites. The incidence is higher in rectal cancer due to venous drainage patterns, that is considered a serious risk factor (Ilyas, 2024; Petrella et al., 2024). Prognosis is variable, since patients with isolated, respectable lung metastases may have improved survival rates, especially after metastasectomy (Beckers et al., 2021), with some studies reporting 5-year survival rates over 50% after curative resection (Guo et al., 2025; Dahlberg et al., 2025). Standard treatment includes a combination of systemic and local therapies like surgery, ablation, radiation (Beckers et al., 2021; Han et al., 2022). Most patients, however, are not candidates for curative surgery, and the overall 5-year survival for metastatic CRC remains below 20% (Petrella et al., 2024) which reinforces the application of bioengineered metastatic models for better prediction of response to treatment strategies.

The basic factors facilitating the process are the physical properties of the pulmonary system and the less oxidative environment that may support the survival of cancer cells. The lungs serve not only as respiratory organs but also as important regulators of the acid-base homeostasis by controlling drug and toxin metabolism. As they are in contact with both oxygen and toxins their defense mechanism against ROS is disabled by metastatic cancer cells which manage to resist oxidative stress (Valavanidis et al., 2013). Additionally, there are plenty of mitochondria and active enzymes that reinforce energy production by metabolizing carbohydrates, fats and proteins (Alvarado and Arce, 2016). All these factors turn the lungs into an attractive target for tumour cells.Essential role in lung metastasis is contributed to the metabolic reprogramming and the nutrients available in the target organ where migrating cancer cells reside. Recently, it has been shown that pulmonary aspartate triggers a cellular signaling cascade in disseminated cancer cells (Liang et al., 2024). As a result, translational reprogramming follows that enhances the aggressiveness of lung metastases (Doglioni et al., 2025). The same authors have detected high concentrations of aspartate in the lung interstitial fluid of breast cancer patients which activates a specific receptor in cancer cells. Lipid metabolism is considered essential for the pathogenesis of CRC metastasis in the lungs and the resistance to oxidative stress (He et al., 2023). The overexpression of ATP-citrate lyase (ACLY), a protein in the initial rate-controlling step of lipid synthesis, and of stearoyl-CoA desaturase 1 (SCD1) are shown to be involved in CRC lung metastasis by promoting the EMT (Ran et al., 2018; Wen et al., 2019). Moreover, the reprograming cellular glycolytic metabolism increases the antioxidant capacity, which facilitates the metastasis of CRC. By reworkinng cancer metabolism the activated Glut3-YAP signaling pathway directs lung-preferred metastasis of CRC cells. It enhances lung colonization via increased cancer cell metabolic reprogramming (Kuo et al., 2019). Another research showed the link between metastasis and the metabolic enzyme PHGDH, which is overexpressed in triple-negative breast cancer and in melanoma. The catalytic activity of the enzyme facilitates tumour cell proliferation while its low expression supports cancer dissemination and metastasis including in the lungs. Therefore, the authors assume that PHDGH heterogeneity in primary tumours may be considered a sign of tumour aggressiveness (Rossi et al., 2022).

Being quite frequent and difficult to cure lung metastases are a serious challenge in oncology which demands novel therapeutic approaches. Tissue engineering and 3D bioprinting offer new possibilities to mimic the complex architecture of the organ and to study the metastatic process with the final aim of providing successful treatment (Imparato et al., 2022). The organ-on-a-chip technology has contributed to the understanding of human lung structure-function relationships at different levels but there are very few investigations on modelling lung metastases (Bai and Ingber, 2022). Despite the high preliminary expectations, lung-on-chips using polydimethylsiloxane (PDMS) artificial elastic membranes could not mimic the alveolar basal membrane as composition and mechanical properties. Shen et al. replaced the PDMS film by a stretchable membrane based on F127-DA hydrogel which possessed a similar composition and stiffness of the ECM of human alveoli (Shen et al., 2023). This model reproduced normal epithelial and endothelial functions, and alveolar-capillary barrier. It also may serve as a platform to explore fibrotic lung diseases and lung metastases.

Recently, Nairon et al. reported the production of thyroid-to-lung metastasis-on-a-chip model which permits invasion analysis and quantification on a single cell level (Nairon et al., 2024). Cells are circulated through microfluidic channels, running parallel to lung hydrogel constructs allowing tumour cell-lung tissue interactions. This innovative model system is validated and helps to evaluate and quantify the adherence and invasion of thyroid cancer cells migrating to the lungs. It could be used to perform in depth mechanistic studies of the metastatic pathway in the lungs. Furthermore, new perspectives for translational research and drug assessment targeting lung metastases could be developed.

Using soft lithography, Kwak and Lee fabricated a vascularized tumour model for organ-specific cancer metastasis. Human microvascular blood endothelial cells and target parenchymal organ cells are introduced into engineered blood vessels to mimic the ECM of the target organs followed by seeding of breast cancer cells with specific metastatic properties. This model allows to follow the extravasation of breast tumours to the lung (but also to the bone as the most common site of metastasis) (Kwak and Lee, 2020). Another group engineered a multi-site metastasis-on-a-chip microphysiological system for assessing metastatic preference of cancer cells. They grew CRC organoids residing in a single microfluidic chamber connected to multiple downstream chambers containing liver, lung, and endothelial constructs. Following the recirculating fluid flow, tumour cells preferentially migrate to the liver and lung constructs as tracked by fluorescent imaging (Aleman and Skardal, 2019). These are the organs to which CRC metastases most commonly occur in human patients, which is an indication that this model could serve as a tool to identify and target metastases.

## 4 Summary of current limitations of 3D biotechnologies

For many decades, animal experiments were the gold standard for disease modelling, drug development, and cancer research. This was due to a lack of knowledge regarding the physiology of complex tissues and the absence of technologies for assembling living tissue in 3D. *In vitro* techniques were also predominantly based on 2D cell culture with tumour cell lines. For many years, human cell lines were selected for efficient growth in normoxic conditions using cell culture media that contained high concentrations of growth factors, such as those found in foetal calf serum. This prioritised growth over physiological function, limiting the relevance of human cell lines for disease modelling and drug testing. In respect to animal experiments, significant interspecies differences, such as those between rodents and humans, were disregarded, implying that if a drug demonstrates efficacy or if a physiological process such as metastasis can be replicated in mice, this must also be valid for humans. However, the fact that more than 90% of “animal-validated” drugs fail in clinical trials suggests otherwise. In the context of drug development, it is also important to note that the reverse is true: compounds that are ineffective in experimental animals will not be developed further. This means that potentially valuable therapeutics are excluded from development due to failure in animals that diverged from humans over 100 million years ago.

Significant progress has been made in stem cell reprogramming, tissue engineering, microfluidics and the development of structured 3D tissue equivalents over the last 2 decades. These advances have provided us with model systems consisting entirely of human cells, assembled in 3D and embedded in matrices that mimic at least partially the structure and mechanical properties of native human tissue. These model systems offer significant advantages over animal experiments, not least because of the “evolutionary gap” between humans and animals.

Engineered human tissues allow for organ-specific therapy and drug testing without the effects of drug conversion or elimination. This is an important advantage over experimental animals because upon injection of a drug candidate into mice, its function may be impaired by cytochrome P450 oxidoreductases in the liver. Alternatively, the drug may simply be eliminated via the kidneys or bound to serum proteins, thereby becoming inactive. In contrast to a living animal, selected human tissues can be combined on one chip to study the off-target effects of drugs on the liver, kidneys and heart, for example, and to directly observe how tumour cells metastasise into the tumour microenvironment and other tissues such as the liver and lungs. This enables researchers to interfere with the different steps of metastasis and thus develop specific drugs for specific tumour types rather than “general” metastasis inhibitors.

However, current *in vitro* models also have limitations: microfluidics typically comprise single cell layers on porous membranes, which do not accurately reflect the 3D context of human organs, with oxygen partial pressures below 20%, or the varying stiffness of human tissues, which significantly impact cell behaviour. While some microfluidics allow mechanical stimulation to mimic mechanobiological processes, they generally fail to reflect many aspects of human tissue. Nevertheless, liver microfluidics have proven to be more effective than rodent toxicity testing at predicting liver toxicity, even leading to a change in FDA approval requirements.

These drawbacks can be partially compensated for, however, with systems that combine fluidic chip devices with 3D scaffolds or structured tissues assembled via 3D bioprinting. These devices significantly extend the ability of microfluidics to mimic tissue-specific stiffness, different matrix compositions and reduced oxygen availability, and thus represent a significant step forward.

Despite progress in developing 3D bioprinted tissue equivalents and organ-on-chip models, limitations regarding the complexity of tissue-on-a-chip models remain. These include the absence of immune components and inefficient blood vessel formation. With respect to the first, different sites of metastasis are populated by distinct local immune cells that contribute to the process. The complex and context-specific role of microglia in the brain, osteoclasts and osteomacs in the bone, Kupffer cells in the liver, alveolar macrophages and other tissue resident macrophages and immune cells have been highlighted by a number of recent review articles (Cao et al., 2024; Bied et al., 2023; Chi et al., 2024). To successfully mimic and study the “seed” and soil” interactions these components would have to be incorporated in future *in vitro* models.

There have been a lot of efforts in this direction (of adding immune components in the systems), albeit in cancer OoCs rather than cancer metastasis models (for which we found only two relevant papers incorporating immune cells as well) and mostly in the context of the intra/extravasation steps of the metastasis cascade, rather than in invasion or colonisation. The importance of macrophages in tumour cell intra/extravasation has been demonstrated by Zervantonakis et al. who used a microfluidic device with endothelial cells in one channel and breast cancer cells in another (the two separated by an ECM-mimicking hydrogel), together with either macrophages or TNFα perfusion. The group detected significantly enhanced extravasation due to macrophages (and paracrine secretion) (Zervantonakis et al., 2012). In line with this, Kim et al. have shown in a similar 2-channel endothelial-tumour cells interface chip that pre-seeding of macrophages can increase vascular permeability. This was found to be achieved by the secretion of metalloproteinases that disrupt endothelial cell tight junctions thus facilitating breast cancer cell intravasation in this model (Kim et al., 2019). Shedding more light on the role of macrophages in the steps of intra/extravasation of tumour cells, Bi et al. compared M1 and M2 polarised macrophages that were perfused together with pancreatic or colorectal cancer cells. While M1 type cells inhibited tumour growth and angiogenesis, M2 macrophages enhanced the migration of cancer cells in the adjacent endothelium-coated channel (Bi et al., 2020). Importantly, Aung et al. have presented compelling evidence that while monocyte-cancer cell co-cultures indeed increase endothelial cell permeability, this is not necessarily an event that would only enhance tumour progression, but it also facilitates T-cell recruitment (perfused in the medium) (Aung et al., 2020). Of note, other immune cells have also been incorporated successfully into heterotypic microfluidic systems such as Kupffer-like cells (THP-1-derived) in an “inflamed” hepatocellular carcinoma model (Ozkan et al., 2023), or NK cells that can migrate from a medium-perfused compartment to a breast cancer compartment and induce tumour cell death (Marzagalli et al., 2022; Ayuso et al., 2019; Ayuso et al., 2021).

With regards to specific efforts focussed on mimicking metastasis to the most common organs and incorporating immune system cells in microfluidic devices, we found only two published works. Lim et al. developed a brain choroid plexus microfluidics device that mimics the capillary-epithelial interfaces by having perfusable areas for both cell types. What their system allowed was to seed breast cancer cells and also flow THP-1 acute myeloid leukaemia cells (mimicking monocytes) that would be polarized to M2 type macrophages by the TME. The research group then assessed the effect of anti-HER2 antibodies alone and in combination with THP-1 cells and observed enhanced cytotoxic effects when both therapeutic approaches were applied (Lim et al., 2023). Crippa et al. have developed a breast cancer to bone metastasis model where breast cancer cells are co-cultured with MSC-derived osteo-differentiated cells, fibroblasts and endothelial cells. This model allowed also the perfusion of primary neutrophils to follow neutrophil extravasation into the breast cancer metastasis niche and the cytotoxic effect of these immune cells on the tumor cells (Crippa et al., 2022).

One of the main reasons for the general absence of immune components and inefficient blood vessel formation is that multiple cell types are needed for both capillary formation and tissue-resident immune cells. It is necessary to optimise the media and matrices for all the cells present in the tissue, and in some cases the growth factors or hormones required by the cells conflict with each other. Therefore, creating a medium and matrix components that meet the requirements of all the cells present, while also steering capillary formation and immune cell differentiation, is one of the major challenges in developing highly complex, human-like tissue equivalents.

## 5 Conclusion

The convergence of microfluidic chip devices and bioprinting will spark the next revolution in disease modelling and our understanding of complex physiological processes in tissues, such as metastasis and immune activation. It will also provide tools for drug development and personalised medicine. Integrating physiological sensors (pH and oxygen) and agitators into the chip, rather than using external pumps, sensors and valves is a promising strategy. The incorporation of sensors that can measure cellular parameters directly, such as cell death (LDH) or specific cytokines, or capture specific circulating cell types, such as activated immune cells or metastatic cells, will provide a wealth of information in ‘real time’ from small tissue chips alone. Connecting multiple organ modules with shared artificial blood media will enable human-on-chip systems to provide a much more complex view of organ crosstalk, (drug)metabolism and cell travel between tissues, including metastasis to distinct tissues. Combining the development of vascularised tissues with dynamic bioinks that respond to physiological stimuli in order to mimic the ECM, as well as modelling tissue at the micron or submicron level using high-resolution printing techniques such as two-photon polymerisation, will enable researchers to create tissues that reflect the complexity of human organs much more accurately than current models do. The result will be highly sophisticated ‘human avatars on a chip’ that can efficiently predict how a patient will respond to drugs and the outcome of therapy.
